# Evaluation of the Expression Profile of Irinotecan-Induced Diarrhea in Patients with Colorectal Cancer

**DOI:** 10.3390/ph14040377

**Published:** 2021-04-19

**Authors:** Mashiro Okunaka, Daisuke Kano, Reiko Matsui, Toshikatsu Kawasaki, Yoshihiro Uesawa

**Affiliations:** 1 Department of Medical Molecular Informatics, Meiji Pharmaceutical University, Kiyose, Tokyo 204-8588, Japan; mokunaka@east.ncc.go.jp; 2 Department of Pharmacy, National Cancer Center Hospital East, Kashiwa, Chiba 277-8577, Japan; dkano@east.ncc.go.jp (D.K.); rmatsui@east.ncc.go.jp (R.M.); tkawasak@east.ncc.go.jp (T.K.)

**Keywords:** diarrhea, irinotecan, fluorouracil, S-1, chemotherapy, spontaneous reporting system, Japanese Adverse Drug Event Report (JADER), pharmacovigilance, time-to-onset analysis, Weibull distribution

## Abstract

Irinotecan (CPT-11) is widely used for the treatment of unresectable colorectal cancer in combination with fluoropyrimidines, such as 5-fluorouracil and S-1. Diarrhea is one of the adverse effects associated with CPT-11 and frequently reported by patients treated with CPT-11-containing regimens combined with oral fluoropyrimidines. However, the mechanisms involved in this process, as well as whether fluctuations in the frequency and differences in the onset time of diarrhea with each CPT-11-containing regimen are caused by drug interactions remain unclear. Therefore, we examined the incidence of diarrhea caused by each CPT-11-containing regimen in patients with colorectal cancer using data from the large voluntary reporting Japanese Adverse Drug Event Report (JADER) database. Firstly, we searched for suspected drugs related to the occurrence of diarrhea using reported odds ratio and calculated the signal score to assess drug–drug interactions. Subsequently, we conducted a time-to-onset analysis using Weibull distribution. The results showed that the combination of CPT-11 with S-1 increased the frequency of diarrhea due to a pharmacological interaction but delayed its onset. The present results may contribute to the appropriate management of drug-induced adverse effects by healthcare professionals.

## 1. Introduction

Colorectal cancer (CRC) remains the second most common cause of cancer-related mortality worldwide. In Japan, irinotecan (CPT-11) is widely used for the treatment of unresectable CRC in combination with fluoropyrimidines, such as 5-fluorouracil (5-FU) and S-1 (Taiho Pharmaceutical Company, Tokyo, Japan). The latter is an oral anticancer drug that combines tegafur with 5-chloro-2,4-dihydropyrimidine (CDHP) and potassium oxonate in a molar ratio of 1.0:0.4:1.0 [1,2].

CPT-11 hydrochloride is a prodrug of 7-ethyl-10-hydroxycamptothecin (SN-38), which is approximately 100- to 1000-fold more cytotoxic than the parent compound. SN-38 prevents DNA recombination by topoisomerase I. As a result, DNA damage is not efficiently repaired and apoptosis ensues due to the induction of replication arrest and DNA double-strand breaks [3,4].

Diarrhea is one of the adverse effects associated with CPT-11 and classified as acute or delayed diarrhea [3,4,5,6,7]. Acute diarrhea is caused by cholinergic reaction. CPT-11 inhibits acetylcholinesterase activity via the 4-piperidinopiperidine moiety during infusion and within a few hours after its administration. The anticholinesterasic effect of SN-38, which is an active metabolite, is weaker than that of CPT-11 (approximately 1/1000). It is considered that SN-38 is not involved in the cholinergic reaction at clinical doses of CPT-11 [5,6,7]. The occurrence of acute diarrhea induced by CPT-11 can be avoided by premedication with atropine monotherapy, which works as a competitive antagonist at anticholinergic receptors. In contrast, delayed diarrhea is attributed to mucosal damage caused by SN-38. Considering the lack of a clear prophylactic treatment, the use of loperamide hydrochloride or octreotide is recommended as a coping therapy in the event of symptoms [5]. When the delayed diarrhea becomes severe, it may lead to dehydration, electrolyte imbalance, and hemodynamic collapse [5]. According to clinical trials, delayed diarrhea is defined as one of the main causes of the dose-limiting toxicity of CPT-11; thus, the management of CPT-11-induced delayed diarrhea is important [4].

It has been shown that the administration of 5-FU and S-1 in combination with CPT-11 also causes diarrhea. 5-FU, an antimetabolite and pyrimidine analog, primarily inhibits thymidylate (dTMP) synthase, which methylates deoxyuridine monophosphate (dUMP) to form thymidine monophosphate (dTMP). The administration of 5-FU depletes dTMP, thereby inducing cell death via thymine deficiency in rapidly dividing cancerous cells. Active metabolites exhibit antitumor activity by inhibiting DNA and RNA synthesis; however, when they act on intestinal mucosal epithelial cells, they cause diarrhea [8].

S-1 is an oral fluoropyrimidine derivative that combines tegafur with two modulators of 5-FU metabolism, namely CDHP (a reversible inhibitor of dihydropyrimidine dehydrogenase) and potassium oxonate. Tegafur, an oral prodrug of 5-FU, is gradually converted to 5-FU and rapidly metabolized by dihydropyrimidine dehydrogenase in the liver. Potassium oxonate is an orotate phosphoribosyl transferase inhibitor, which preferentially localizes in the digestive tract. These components of S-1 decrease the incorporation of 5-FU triphosphate into RNA in the gastrointestinal mucosa and reduce the incidence and severity of diarrhea [9,10].

Previous studies reported that diarrhea occurs especially frequently in patients treated with CPT-11-containing regimens combined with oral fluoropyrimidines [11,12,13]. However, the mechanisms involved in this process, as well as whether fluctuations in frequency and differences in the onset time of diarrhea with each CPT-11-containing regimen are caused by drug interactions remain unclear.

Therefore, we examined the occurrence of diarrhea induced by each CPT-11 regimen in patients with CRC using data from the Japanese Adverse Drug Event Report (JADER) database—a publicly available spontaneous reporting database created by the Pharmaceuticals and Medical Devices Agency (PMDA). This database has been recognized as one of the tools for pharmacovigilance assessments. The JADER database contains a portion of Japanese ADR reports released from April 2012 to September 2020. Analysis of time-to-onset data and a subset analysis to screen for drug–drug interactions have been proposed for the detection of adverse drug reaction signals in a spontaneous reporting database.

## 2. Results

### 2.1. Presentation of Data

The drug information (DRUG) table included 3,567,277 cases, while the adverse event (REAC) table included 1,218,252 cases. The total number of cases, as shown in the “all data table”, was 20,174,288. The “suspected medicine data table”, which only extracted suspected medications from the all data table, included a total of 6,182,184 cases.

### 2.2. Diarrhea and Patient Background

In the “suspected medicine data table”, diarrhea was reported in 8835 patients treated with CPT-11. Table 1 shows the characteristics of patients treated with CPT-11 who reported adverse events (diarrhea or others). *p*-values were calculated using the Fisher’s exact test. The occurrence of diarrhea and non-diarrhea was significantly more frequent in males. Differences in age did not affect the development of diarrhea.

### 2.3. Diarrhea-Inducing Medications

**Figure 1:**
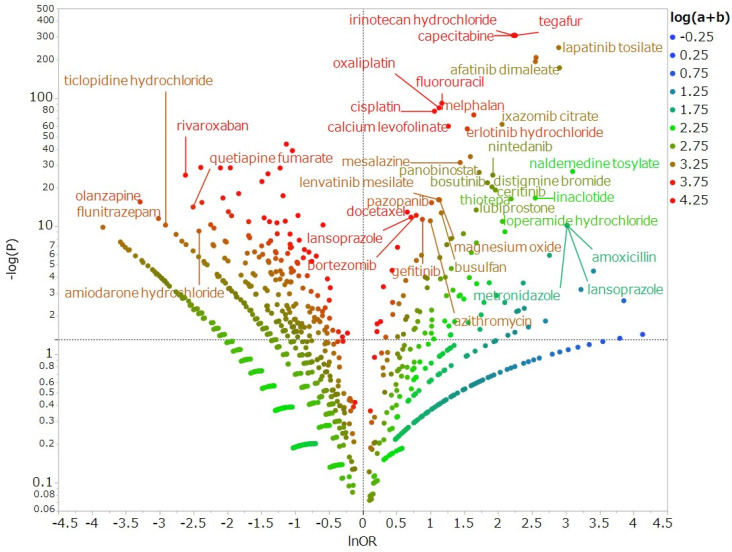
Drugs associated with the development of diarrhea. The X-axis shows natural logarithm of odds ratio (lnOR) values, whereas the Y-axis shows common logarithm of inverse *p*-values (−log10 (*p*)) obtained by Fisher’s exact test. The dotted line on the Y-axis represents a *p*-value of 0.05. Plot colors represent the number of reported adverse events. Blue, green, and red dots are common logarithms of total reported numbers (from −0.25 to 4.25).

To examine drugs suspected of causing diarrhea and other drugs, we exhaustively produced a volcano plot, which is frequently employed for the visual representation and understanding of trends in genetic expression in the field of bioinformatics (Figure 1). As shown by the positive logarithmically transformed odds ratios (lnOR) represented on the X-axis, drugs located in the positive direction on the X-axis indicated that diarrhea was reported more frequently than other adverse events. As indicated by the higher values of logarithmically transformed inverse *p*-values on the Y-axis, drugs are shown that the higher positive value on the Y-axis indicated a strong significant difference. In other words, the reported odds ratio (ROR) and significant difference were more likely to induce medication-related diarrhea drawn in the upper right side. The results of the volcano plot showed that the anticancer drugs capecitabine, lapatinib, and afatinib were associated with the development of diarrhea. CPT-11, 5-FU, and S-1 were selected for this investigation drawn in the upper right side and shown to be significantly associated with diarrhea (*p* < 0.001 for all).

### 2.4. Diarrhea as a Result of Drug–Drug Interaction

The RORs for diarrhea induced by CPT-11, 5-FU, and S-1 in monotherapy were 9.258 (95% confidence interval (CI): 8.633–9.928), 3.211 (95% CI: 2.918–3.534), and 9.511 (95% CI: 8.900–10.164), respectively. In addition, the RORs for diarrhea induced by combination therapy of these drugs (CPT-11 plus 5-FU and CPT-11 plus S-1) were 6.199 (95% CI: 5.260–7.306) and 25.695 (95% CI: 22.042–29.954), respectively (Table 2). Next, we examined whether concomitant therapy with fluoropyrimidine chemotherapy increased the risk of diarrhea in patients treated with CPT-11 using the subset data. The subset data were used to assess drug–drug interactions. Subset analysis detected both S-1 or CPT-11 diarrhea signals in the CPT-11 or S-1 patient group, respectively. The results showed that the combination of CPT-11 and S-1 increased the risk of diarrhea as drug-drug interaction (Table 3). Of note, the lower limit of the 95% CI of the 5-FU signal in patients using CPT-11 was ≤1 (ROR: 0.442; 95% CI: 0.398–0.491) (Table 4). Therefore, there were no significant associations observed between diarrhea and CPT-11 with 5-FU.

### 2.5. Time of Onset of Diarrhea

**Figure 2:**
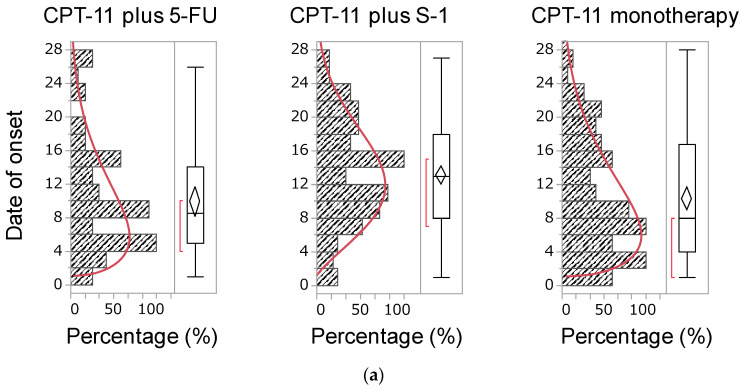

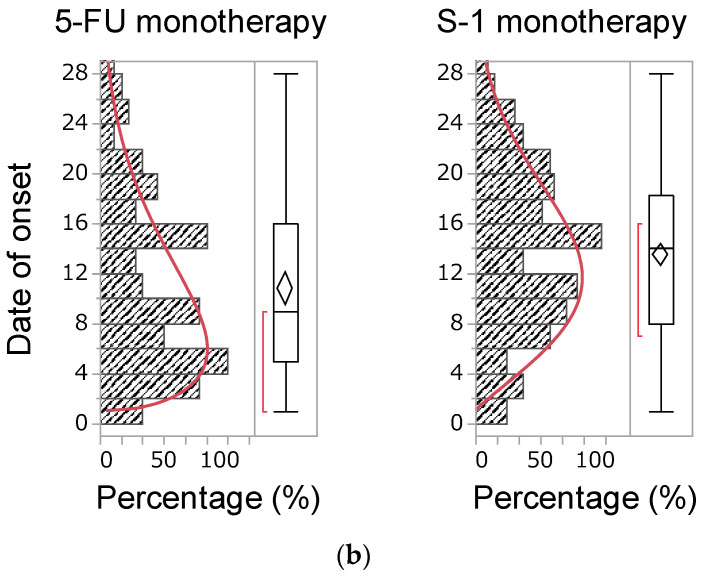
(**a**) Histogram and the Weibull shape parameter of the time of onset of diarrhea by an irinotecan (CPT-11)-containing regimen. The X-axis shows the percentage of the maximum frequency of diarrhea. The maximum frequency of diarrhea was 11, 22 and 19 for CPT-11 plus 5-FU, CPT-11 plus S-1 and CPT-11 monotherapy, respectively. The Y-axis shows the number of days of initial diarrhea after administration of the suspected medicine. The red line shows the Weibull shape parameter of diarrhea. (**b**) Histogram and the Weibull shape parameter of the time of onset of diarrhea by 5-fluorouracil (5-FU) or S-1. The X-axis shows the percentage of the maximum frequency of diarrhea. The maximum frequency of diarrhea was 20 and 32 for 5-FU monotherapy and S-1 monotherapy, respectively, whereas the Y-axis shows the number of days of initial diarrhea after administration of the suspected medicine. The red line shows the Weibull shape parameter of diarrhea.

A total of 592 and 622 reports contained data indicating the time of onset of diarrhea induced by CPT-11- and fluoropyrimidine-containing therapies, respectively. The medians of the time of onset of diarrhea caused by a CPT-11-containing regimen were as follows: 8.5, 14.0, and 8.0 days for CPT-11 plus 5-FU, CPT-11 plus S-1, and CPT-11 monotherapy, respectively. Moreover, these values for 5-FU and S-1 were 9.0 and 14.0 days, respectively (Figure 2). Patients treated with the CPT-11 plus S-1 regimen developed diarrhea later than those who received other CPT-11-containing regimens. In addition, with fluoropyrimidine monotherapy, S-1 was linked to the development of significantly delayed diarrhea versus 5-FU (Table 5). The median duration and cumulative incidence rate were used to evaluate the data regarding time-to-onset of diarrhea. The cumulative incidence rate for each drug was determined using the Kaplan–Meier method, and the time-to-onset profiles were analyzed using the Weibull shape parameter test. The cumulative incidence of diarrhea in CPT-11-containing regimens showed that CPT-11 monotherapy and the combination of CPT-11 and S-1 were associated with the earliest and latest onset of diarrhea, respectively. The Weibull distribution parameters for all chemotherapy regimens are summarized in Table 6. The shape parameter β determines the shape of the distribution function, which corresponds to the development of an adverse reaction: when β < 1 (early failure type), the incidence decreases with time; when β = 1 (random failure type), adverse reactions develop at a constant rate; and when β > 1 (wear-out failure type), the incidence decreases with time [14]. The shape parameter β of all treatments showed a lower limit of 95% CI > 1, suggesting a profile of the wear-out failure type. A comparison of fluoropyrimidines showed that S-1 monotherapy (β = 2.075, 95% CI: 1.855–2.309) was associated with the development of more delayed diarrhea than 5-FU monotherapy (β = 1.511, 95% CI: 1.265–1.782) (Table 6).

## 3. Discussion

### 3.1. Diarrhea as a Result of Drug–Drug Interaction

This is the first report to investigate the incidence of diarrhea, causality of drug–drug interaction, and timing of onset induced by various CPT-11-containing regimens. Our study yielded two important findings regarding CPT-11-induced diarrhea. The first major finding is that the combination of CPT-11 plus S-1 increases the incidence of diarrhea due to a pharmacological interaction. Since intestinal mucosal epithelial cells are actively dividing and proliferating, they are susceptible to damage from anticancer drugs and radiation. Damage to intestinal mucosal epithelial cells is the main cause of diarrhea [5]. CPT-11 and fluorinated pyrimidines are anticancer agents administered in the treatment of CRC and are likely to cause diarrhea [15]. CPT-11, 5-FU, and S-1 were plotted in the upper right of the volcano plot, indicating that these drugs caused diarrhea more frequency than other anticancer agents. Diarrhea is caused by CPT-11 and SN-38 through enhanced cholinergic intestinal peristalsis and direct damage to the gastrointestinal mucosa, respectively. 5-FU and tegafur (actual chemotherapeutic agent of S-1) cause diarrhea through DNA disorders (due to dTMP synthase inhibition to 5-fluorouridine 5′-triphosphate incorporated into RNA). In addition, the gimeracil and oteracil potassium contained in S-1 do not have cytotoxic effects on intestinal mucosal epithelial cells. Hence, the main cause of diarrhea after treatment with S-1 is considered to be the action of tegafur [9,16]. Thus, since CPT-11, 5-FU, and S-1 act on similar sites in the intestinal mucosal epithelial cells, it is considered that both drugs cause diarrhea. However, there were no reports focusing on the interaction between CPT-11 and 5-FU or S-1. The observation that the combination of CPT-11 plus S-1 increases the incidence of diarrhea due to a pharmacological interaction is a new finding. Previous reports have reported that the pharmacokinetics of CPT-11, SN-38, and SN-38G are not affected by the oral administration of S-1 in the CPT-11 plus S-1 regimen [17]. Nevertheless, it has been reported that the CPT-11 plus 5-FU regimen reduces the AUC of SN-38 due to the effects of 5-FU after the administration of CPT-11 [9,17]. This suggests that the CPT-11 plus 5-FU regimen is associated with milder diarrhea compared with CPT-11 monotherapy and the CPT-11 plus S-1 regimen.

### 3.2. Time of Onset of Diarrhea

The second major finding of this study suggests that the CPT-11 plus 5-FU regimen is more likely to cause diarrhea later than other CPT-11-containing regimens. CPT-11-induced diarrhea is classified as early onset (occurring within 24 h after administration) and late-onset (occurrence peaking 4–10 days after administration) [5]. In contrast, fluoropyrimidine-induced diarrhea is thought to develop between 1 week and 2 months after infusion [18,19]. Generally, the schedule of the CPT-11 plus S-1 regimen includes the administration of CPT-11 on day 1 and continuous administration of S-1 for 2 weeks (days 1–14) [3,4]. Therefore, it is considered that diarrhea was prolonged by the CPT-11 plus S-1 regimen compared with that induced by other CPT-11-containing regimens due to intestinal mucosal damage caused by S-1. In this study, it was revealed that S-1 monotherapy causes diarrhea later than 5-FU monotherapy. Based on this result, it is necessary to monitor the side effects caused by the CPT-11 plus S-1 regimen, with particular attention to the onset of delayed diarrhea, considering the effects of S-1 used in combination.

### 3.3. Limitations

The JADER database is based on a spontaneous reporting system, and the reported cases were limited by the recognition of side effects. First, mild adverse effects are only occasionally reported whereas severe adverse effects are reported more often. This is a known reporting bias, which is characteristic of self-reporting databases [20]. Second, this database is problematic, given that the association between medicines and adverse events was not clear [21]. If several drugs are concomitantly administered to a single patient, it is difficult to identify the causes of an adverse drug reaction [22,23,24,25]. Third, the cases reported in the JADER database included true as well as suspected adverse effects because the reports were based on personal judgment.

Therefore, it is important to compare detection trends using all adverse events recorded in the validation dataset that is created based on a spontaneous reporting system; however, it takes an extremely long time to calculate signal values for all combinations of drug–drug interactions. Therefore, we performed the targeted analysis of CPT-11, 5-FU, and S-1. In addition, confounding background factors, such as sex differences, which were significantly associated with the onset of diarrhea, were not investigated in this study. Therefore, the analysis of JADER database alone cannot completely determine the contribution of 5-FU and S-1 to CPT-11-induced diarrhea and further investigation is necessary to clarify the exact time of onset and the drug–drug interactions.

## 4. Materials and Methods

### 4.1. Database Information

Since April 2004, the PMDA has reported cases of adverse events associated with medications in the JADER database [26]. JADER is a large-scale database that contains information on the adverse effects of medications and patients in Japan. We accessed the JADER database from the PMDA website and performed our analysis using data reported between April 2004 and September 2020.

### 4.2. Production of a Data Analysis Table

**Figure 3:**
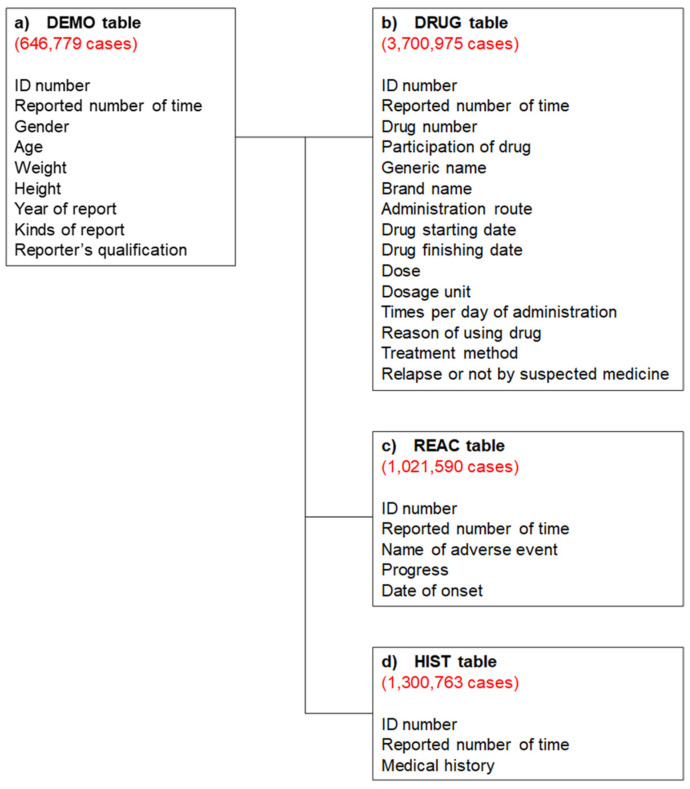
Four information tables included in the Japanese Adverse Drug Event Report (JADER) database. The red numbers show the number of reports obtained between April 2004 and September 2020.

**Figure 4:**
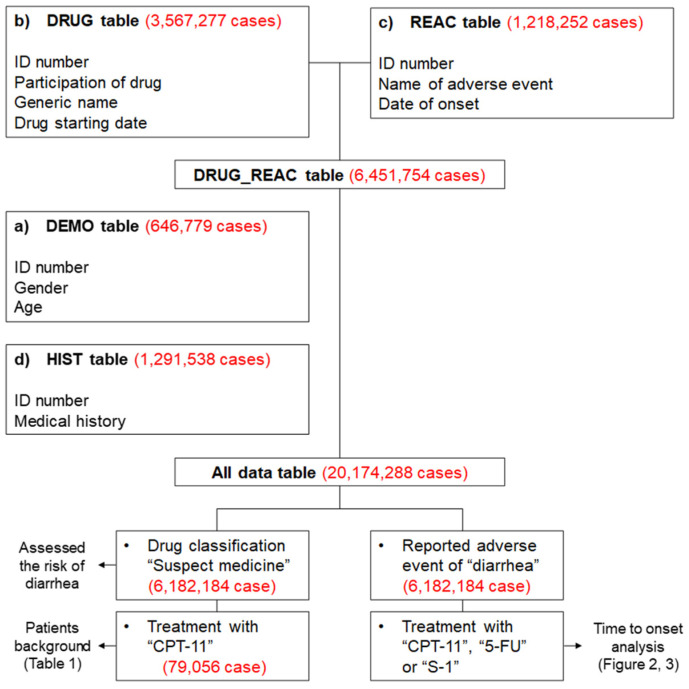
Flow chart for the construction of data analysis tables. The DRUG table (drug name, causality, etc.) was classified into three categories: “suspected medicine”, “concomitant medicine”, and “interaction medicine.” We extracted all information relevant to these categories from the DRUG table. We removed duplicated data from the DRUG and REAC tables (adverse events, outcome, etc.). Data in the DEMO table (patient demographic information, such as sex and age) and HIST table (medical history) were combined with the DRUG and REAC tables using patient identification numbers. Based on the combined table, only “suspected medicine” information was used to assess the risk of diarrhea. All information on “suspected medicine”, “concomitant medicine”, and “interaction medicine” were used for the time-of-onset analysis.

All data were analyzed to investigate the patient’s background and the drug that caused the diarrhea. The JADER data consist of four tables: (a) patient demographic information (DEMO); (b) DRUG; (c) REAC; and (d) medical history (HIST). These four tables were used for all analyses in this study (Figure 3). Duplicated data were removed from the DRUG and REAC tables. Next, the removed duplicated data were linked using patient identification numbers to create the DRUG_REAC table. Subsequently, the DEMO and HIST tables were merged to create the DRUG_REAC table using patient identification numbers. This table was termed the “all data table” (Figure 4). In each case, the contribution of given medications to the adverse events was classified into three categories: “suspected medicine”, “concomitant medicine”, and “interaction.” We extracted all cases classified according to these categories for analysis.

### 4.3. Analyzed Drugs and Adverse Event Terms

Three chemotherapeutic, CPT-11-containing regimens were selected for this investigation: CPT-11 monotherapy; CPT-11 plus 5-FU; and CPT-11 plus S-1. We also analyzed S-1 monotherapy and 5-FU monotherapy for comparison. The adverse event terms appearing in the JADER database are based on the High Level Group Term, High Level Term, and Preferred Term in the Medical Dictionary for Regulatory Activities/Japanese version (MedDRA/J) [27]. For the analysis of diarrhea, we extracted four Preferred Terms (i.e., diarrhea, diarrhea hemorrhagic, diarrhea neonatal, post-procedural diarrhea), which are classified as diarrhea (excluding infective) (High Level Term: 10012736) in the MedDRA/J version 23.0.

### 4.4. Relationship Between Suspected Medicines and Diarrhea

We assessed the risk of diarrhea for each drug using RORs and Fisher’s exact test. Initially, we created a 2 × 2 contingency table of drugs and side effects for each drug (Table 7). The 2 × 2 contingency table cannot be calculated with zero cells, and the estimation is unstable when the cell frequency is small. Therefore, 0.5 was added to all cells as a correction (Haldane Anscombe half correction) [28,29]. In this study, diarrhea-related drugs were defined as those with a ROR ≥ 1 and a Fisher’s exact test *p*-value < 0.05 [30]. Subsequently, a scatterplot consisting of RORs and *p*-values was created for a visual interpretation of the drug effects. This scatter plot was created by converting RORs to lnORs and the *p*-values obtained from the Fisher’s exact test to common logarithms (−log10(*p*-value)). The scatter plot corresponds to volcano plots frequently used to understand gene expression trends in bioinformatics [31,32]. Furthermore, the ROR was used to calculate the signal score for assessing drug–drug interactions. In this algorithm, signals for drug D2 or D1 detected in the subset of a patient group using drug D1 or D2 were indicative of drug–drug interaction [33].

The cross-tabulation is structured with reports for the suspected drug, all other reports, reports with adverse events, and reports without adverse event (a–d indicate the number of reports).

### 4.5. Onset Time of Diarrhea

In the analysis of the time-to-onset of adverse events, only data for patients who reported diarrhea after receiving CPT-11, 5-FU, or S-1 were extracted. The number of days until the development of diarrhea was calculated from the date of initial administration of the drug reported and the date of the onset of diarrhea reported in the JADER database. Unknown Reports with missing information on the date of treatment initiation or the onset of adverse events were excluded. In this study, we analyzed >100 reported cases treated with CPT-11-containing regimens. The number of days until the onset of adverse events was calculated by subtracting the date of initiation drug administration from that of the onset of adverse events. A value of 1 was added to the calculated number of days because the initiation date of drug treatment was set as day 1. The upper limit of time-to-onset of adverse events was set at 29 days. This is because, in Japan, the CPT-11-containing regimens usually involve a treatment cycle of 1–4 weeks [7]. The Weibull shape parameter test is used to analyze time-to-onset data and can describe the non-constant rate of the incidence of adverse events [34]. Time-to-onset analysis using the Weibull distribution was performed, and the Weibull parameters α and β were calculated using the JMP^®^ Pro14 (SAS Institute Inc., Cary, NC, USA) software. The shape parameter β of the Weibull distribution represents the failure rate distribution against time. The failure rate corresponds to the development of an adverse reaction: when β < 1 (early failure type), the incidence decreases with time; when β = 1 (random failure type), adverse reactions develop at a constant pace; and when β > 1 (wear-out failure type), the incidence decreases with time [14].

### 4.6. Statistical Analysis

The means (±standard deviation) were calculated for all continuous variables. *p*-values < 0.05 denoted statistical significance. We estimated the internal correlation using the pairwise method. Internal correlation was indicated by a value of the square of Spearman’s rank-order correlation coefficient (*ρ*^2^) > 0.9. In the absence of internal correlation, we treated these items as independent factors. All analyses were performed with the JMP^®^ Pro14 (SAS Institute Inc., Cary, NC, USA) software.

## 5. Conclusions

Through the analysis of a large data set, we were able to determine the incidence of diarrhea in patients treated with CPT-11-containing regimens. Our analyses revealed two important findings. First, CPT-11 used in combination with S-1 was associated with increased incidence of diarrhea due to pharmacological interaction. Second, CPT-11 may be considered for use in combination with S-1 to delay the onset of diarrhea. The present findings highlight the importance of carefully monitoring patients receiving treatment with CPT-11 in combination with S-1. Further verification and investigation of the underlying mechanism in future studies are expected to significantly contribute to the detailed understanding of the interactions revealed in this study.

## Figures and Tables

**Table 1:** Characteristics of patients with or without diarrhea.

Characteristics	Diarrhea		Non-Diarrhea		*p*-Value
	*n*	(%)	*n*	(%)	
Sex (Male/Female) *	534/347	(60.6/39.4)	3,674/2,149	(63.1/36.9)	0.016
Age **	872	65.6 ± 0.42	5,732	64.7 ± 0.16	0.076

Each item includes some missing values. Analyses performed using data after eliminating these records. Numbers in parentheses are the numbers of causes used in the analyses. *: Fisher’s exact test; **: *t* test.

**Table 2:** Reporting odds ratio of diarrhea associated with irinotecan (CPT-11)-containing chemotherapy regimens.

Drug	Reporting Times	Odds Ratio	(95% CI)
CPT-11 monotherapy	11,589	9.258	(8.633–9.928)
5-FU monotherapy	15,118	3.211	(2.918–3.534)
Tegafur, Gimeracil, Oteracil Potassium (S-1) monotherapy	12,697	9.511	(8.900–10.164)
CPT-11 plus 5-FU	1,993	6,199	(5,260–7,306)
CPT-11 plus S-1	866	25,695	(22,042–29,854)

“Reporting times” shows the number of cases that reported a medicine suspected as a cause of diarrhea.

**Table 3:** Diarrhea induced by irinotecan (CPT-11) and S-1 due to drug–drug interaction.

Regimen	Signal for CPT-11		Signal for S-1	
	N	ROR (95% CI)	N	ROR (95% CI)
patients group using CPT-11	–	–	1690	2.042 (1.825–2.284)
patients group using S-1	1690	1.989 (1.780–2.222)	–	–

“Signal for CPT-11” shows diarrhea signal in the CPT-11 patients group taking S-1. “Signal for S-1” shows diarrhea signal in the S-1 patients group receiving CPT-11.

**Table 4:** Subset analysis for the detection of diarrhea induced by irinotecan (CPT-11) and 5-fluorouracil (5-FU) due to a drug–drug interaction.

Regimen	Signal for CPT-11		Signal for 5-FU	
	N	ROR (95% CI)	N	ROR (95% CI)
patients group using CPT-11	–	–	3813	0.442 (0.398–0.491)
patients group using 5-FU	3813	2.091 (1.858–2.353)	–	–

“Signal for CPT-11” shows a signal for diarrhea in the CPT-11 patient group receiving 5-FU. “Signal for 5-FU” shows a signal for diarrhea in the 5-FU patient group receiving CPT-11.

**Table 5:** Median onset of diarrhea induced by chemotherapy regimens.

Regimen	*n*	Time of Onset (Day)		Regimen	*n*	Time of Onset (Day)		*p*-Value *
		Median	Range			Median	Range	
CPT-11 plus S-1	117	14.0	1–27	CPT-11 plus 5-FU	36	8.5	1–26	0.005
CPT-11 monotherapy	112	8.0	1–28	CPT-11 plus 5-FU	36	8.5	1–26	0.911
CPT-11 monotherapy	112	8.0	1–28	CPT-11 plus S-1	117	14.0	1–27	<0.005
5-FU monotherapy	91	9.0	1–28	S-1 monotherapy	223	14.0	1–28	<0.005

The median and statistical significance of the onset of diarrhea with each regimen is shown. *: Wilcoxon test.

**Table 6:** Distribution rate related to diarrhea using the Weibull distribution.

Regimen	*n*	Shape Parameter: β	
		β	95% CI
CPT-11 plus 5-FU	36	1.554	1.179–1.978
CPT-11 plus S-1	117	2.208	1.891–2.551
CPT-11 monotherapy	112	1.461	1.251–1.690
5-FU monotherapy	91	1.511	1.265–1.782
S-1 monotherapy	223	2.075	1.855–2.309

“Median time of onset” shows the time of the onset of diarrhea for each chemotherapeutic agent.

**Table 7:** Cross-tabulation and calculation formula for RORs of adverse events.

	Diarrhea	Non-Diarrhea
Reports with the suspected medicine	a	c
All other reports	b	d

ROR (reporting odds ratio) = (a/b)/(c/d) = ad/bc.

## Data Availability

The data presented in this study are available on request from the corresponding author.
